# HMEJ-mediated efficient site-specific gene integration in chicken cells

**DOI:** 10.1186/s13036-019-0217-9

**Published:** 2019-11-21

**Authors:** Long Xie, Juanjuan Sun, Lifen Mo, Tianpeng Xu, Qaisar Shahzad, Dongyang Chen, Wenhao Yang, Yuying Liao, Yangqing Lu

**Affiliations:** 1 State Key Laboratory for Conservation and Utilization of Subtropical Agro-bioresources, Guangxi University, Nanning, Guangxi China; 2 Guangxi Institute of Animal Science, Nanning, Guangxi China

**Keywords:** HMEJ, Transgenic chicken, CRISPR/Cas9, PGCs, Ovalbumin

## Abstract

**Background:**

The production of transgenic chicken cells holds great promise for several diverse areas, including developmental biology and biomedical research. To this end, site-specific gene integration has been an attractive strategy for generating transgenic chicken cell lines and has been successfully adopted for inserting desired genes and regulating specific gene expression patterns. However, optimization of this method is essential for improving the efficiency of genome modification in this species.

**Results:**

Here we compare gene knock-in methods based on homology-independent targeted integration (HITI), homology-directed repair (HDR) and homology mediated end joining (HMEJ) coupled with a clustered regularly interspaced short palindromic repeat associated protein 9 (CRISPR/Cas9) gene editing system in chicken DF-1 cells and primordial germ cells (PGCs). HMEJ was found to be a robust and efficient method for gene knock-in in chicken PGCs. Using this method, we successfully labeled the germ cell specific gene *DAZL* and the pluripotency-related gene *Pou5f3* in chicken PGCs through the insertion of a fluorescent protein in the frame at the 3′ end of the gene, allowing us to track cell migration in the embryonic gonad. HMEJ strategy was also successfully used in Ovalbumin, which accounts for more than 60% of proteins in chicken eggs, suggested its good promise for the mass production of protein with pharmaceutical importance using the chicken oviduct system.

**Conclusions:**

Taken together, these results demonstrate that HMEJ efficiently mediates site-specific gene integration in chicken PGCs, which holds great potential for the biopharmaceutical engineering of chicken cells.

## Introduction

Gene modification technologies utilized in chicken have great potential for applications in fields such as developmental biology and biomedical research. The production of protein “drugs” via the use of transgenic animals is an emerging field for the pharmaceutical industry. This requires the integration of a desired gene specific to a protein of interest into the genome of recipient animals, making its economically significant expression inherited in successive generations of animals [1]. Traditionally, the introduction of foreign genes into the chicken genome has been achieved using lenti-virus or plasmid vectors [2–5]. This can, unfortunately, lead to the silencing of nearby genes due to random or multi-copy insertions [6, 7]. Furthermore, the expression of foreign genes driven by strong promoters and inserted into the chicken genome could result in unpredictable consequences in tissues or even in whole animals [8].

Site-specific gene integration can be a helpful strategy for avoiding random or multi-copy insertions during the introduction of foreign DNA. In this strategy, the foreign gene is inserted at a targeted position that has minimal influence on the genomic structure or protein expression of nearby genes, as compared to that with a traditional transgene [9–11]. The application of site-specific gene integration can also help with the conditional expression of foreign proteins, as the utilization of endogenous regulators is possible [12, 13].

Many alternative methods have been used to improve the process of site-specific gene integration in chicken cells, including zinc finger nucleases (ZNFs) [14, 15] and transcription activator-like effector nucleases (TALENs) [16]. These methods, however, require complex designs or have been shown to have variable editing efficiency. In recent years, CRISPR/Cas9 (clustered regularly interspaced short palindromic repeat/CRISPR-associated protein 9), which is derived from bacteria, has been demonstrated to be a simple and efficient gene editing tool in yeast and vertebrates [17–19].

Site-specific foreign gene integration is typically achieved through homology-directed repair (HDR), and requires that the gene of interest be flanked by homology arms of approximately 800–6000 bp, with integration only occurring in dividing cells [20, 21]. However, HDR is not readily accessible to non-dividing cells [22]. By contrast, non-homologous end joining (NHEJ) is active in both dividing and non-dividing cells [23]. Homology-independent targeted integration (HITI) was developed based on the events of NHEJ and has been demonstrated to be a robust method for targeted integration of transgenes in both proliferating and post-mitotic cells [24]. Additionally, a strategy termed homology-mediated end joining (HMEJ), which utilizes both HDR and double strand break (DSB) repair pathways to achieve gene integration, has also been demonstrated to be an efficient gene knock-in strategy in mice, suggesting it may have utility in other vertebrates [4].

Genetic modification using the CRISPR/Cas9 system has been shown to be feasible in chicken cells [4]. Combined with the use of primordial germ cells (PGCs), which have been reported as an efficient tool for genetic transmission in chickens [25, 26], CRISPR/Cas9 provides a reasonable platform for the production of genetically modified chicken cells for pharmaceutical bioengineering. In this work, in order to optimize the strategy for gene integration in chicken, different gene integration strategies (HITI, HDR and HMEJ) mediated by CRISPR/Cas9 induction of double stranded breaks were compared in chicken DF-1 cells and PGCs. Our research demonstrated that HMEJ was a robust and efficient method for gene knock-in in chicken PGCs.

## Results

### CRISPR/Cas9 induces targeted DNA cleavage in chicken cells

**Fig. 1:**
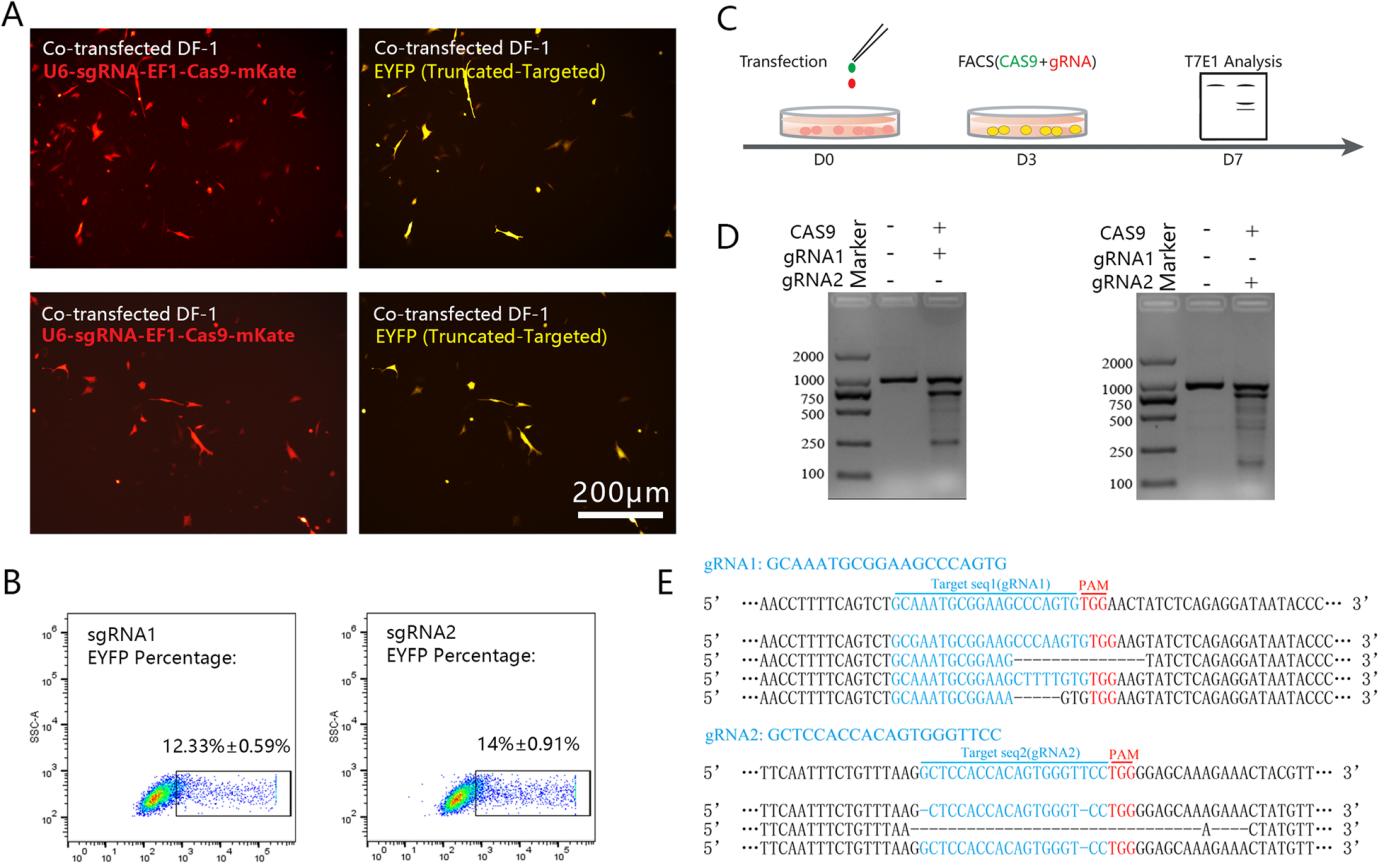
CRISPR/Cas9 induces targeted DNA cleavage in chicken cells. **a** Co-transfection of U6-gRNA-pHEf1A-Cas9-mKate and EYFP-Truncated plasmids in chicken DF-1 (48 h); **b** Flow cytometry analysis of EYFP percentage in co-transfected DF-1 cells in (**a**); **c** Experimental scheme for targeted *DAZL* gene knock out in DF-1 cells; **d** T7 endonuclease I assay of the *DAZL* gene mutation in chicken DF-1 cells; **e** Sequencing analysis of targeted mutation in the *DAZL* gene

The *DAZL* gene in DF-1 cells was targeted to determine the efficiency of the CRISPR/Cas9 system in chicken. Two gRNAs targeting the *DAZL* gene were designed and their targeting sequences were cloned and constructed into a reporter plasmid (EYFP-Truncated-gRNA-EYFP), in which a truncated EYFP protein would be expressed while HDR occurred. EYFP expression was observed in DF-1 cells 48 h after co-transfection of CRISPR/Cas9 plasmids and the reporter plasmid (Fig. 1a and b). This confirmed the cleavage activity of the CRISPR/Cas9 system in chicken cells. To determine the editing efficiency using the CRISPR/Cas9 system, the plasmids pHEf1a-Cas9-2a-EGFP and gRNA-pHEf1a-mKate (gRNA1 and gRNA2) were co-transfected into DF-1 cells, and double positive cells were identified by fluorescent-activated cell sorting (FACS) (Fig. 1c). T7E1 analysis was performed using DNA extracted from sorted cells 7 days after transfection. The mutation efficiency detected by the T7E1 assay was 40% for gRNA1 and 35% for gRNA2 (Fig. 1d). Furthermore, sequencing analysis revealed sense mutations, including point mutations and fragment deletions, at the targeted location (Fig. 1e). These results indicated that efficient site-specific gene editing of CRISPR/Cas9 was possible in chicken cells.

### HMEJ- and HDR-mediated efficient gene integration in chicken somatic cells

**Fig. 2:**
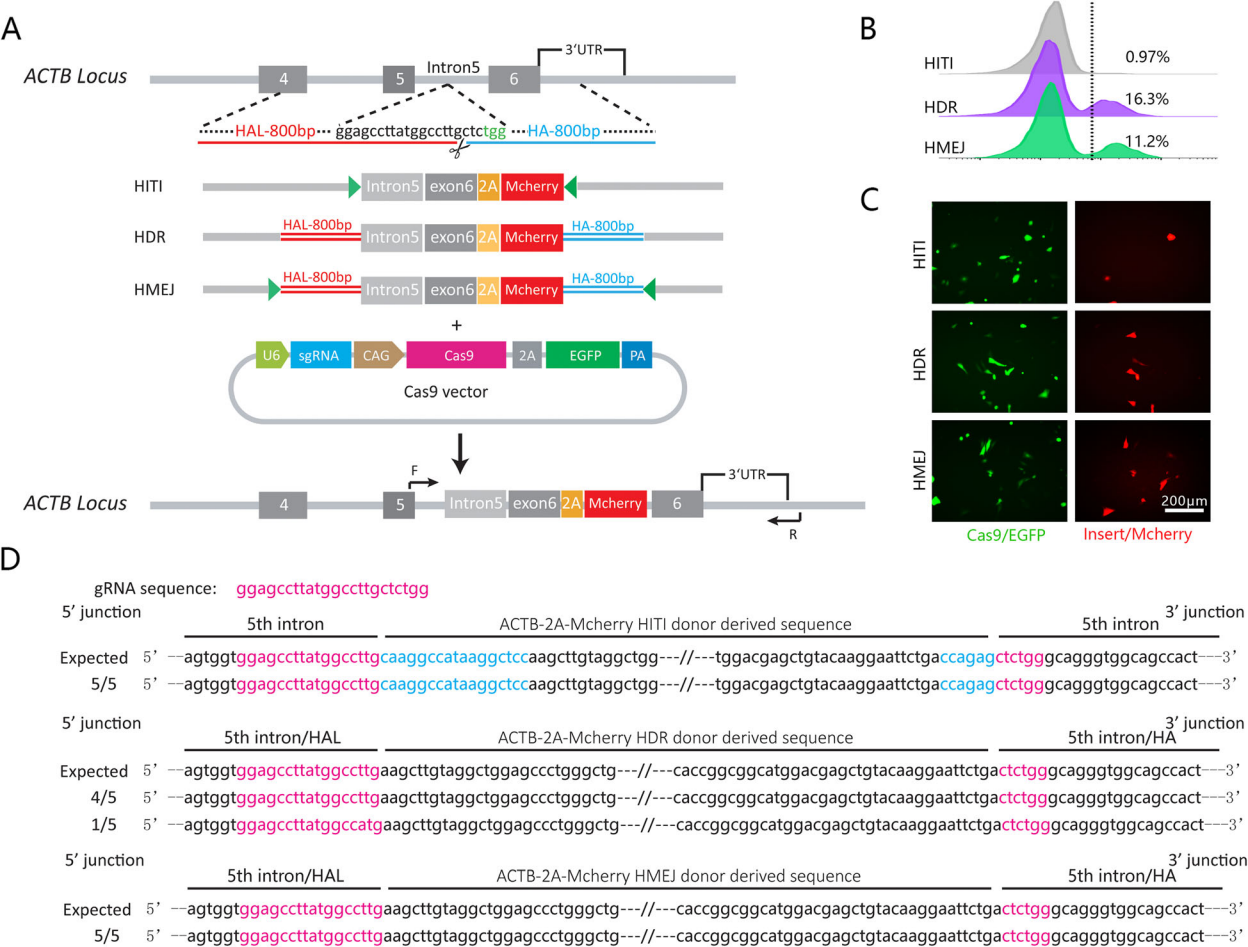
Gene integration in chicken somatic cells mediated by the CRISPR/Cas9 system. **a** Overview of the designs of site-specific gene integration strategies at the chicken *ACTB* locus; **b** Flow cytometry analysis of mCherry (gene integrated) cell proportions using the HITI, HDR and HMEJ strategies; **c** mCherry+ cells were observed using a fluorescence microscope 7 days after co-transfection of Cas9 plasmid and different donor plasmids; **d** Sequencing analysis of site-specific integration meditated by HITI, HDR and HMEJ at the chicken *ACTB* locus

To determine the gene knock-in efficiency when using the CRISPR/Cas9 system, the *ACTB* locus in the chicken genome was targeted and gene integration rates mediated by HITI, HDR and HMEJ were subsequently compared. A gRNA was designed and inserted into a CRISPR/Cas9 plasmid carrying an EGFP component. Off-target analysis of the sgRNA was performed and reveals its specific for *ACTB* (Additional file 1: Figure S1A). A donor plasmid *ACTB*-2A-mCherry was constructed according to the principles of HITI, HDR and HMEJ (Fig. 2a). The CRISPR/Cas9 and donor plasmids were then co-transfected into DF-1 cells in the presence of lipofectamine 3000 and the expression of mCherry was used as an indicator of integration. Three days after transfection, EGFP positive (CRISPR/Cas9 positive) cells were FACS sorted and then cultured for another 4 days until analysis of the gene integration efficiency.

Analysis of integration by flow cytometry showed the knock-in efficiency of HITI, HDR and HMEJ to be 0.97, 16.3 and 11.2%, respectively (Fig. 2b). Sequencing of PCR products amplified from single cells revealed that the integration of donor sequences mediated by HITI, HDR and HMEJ was as expected (Fig. 2d). These results showed that CRISPR/Cas9-mediated efficient gene integration in chicken somatic cells. In addition, these results indicated that a knock-in strategy mediated by homology-arms (HDR and HMEJ) worked better than a non-homologous strategy (HITI) in DF-1 cells.

### HMEJ mediates robust gene integration in chicken PGCs

**Fig. 3:**
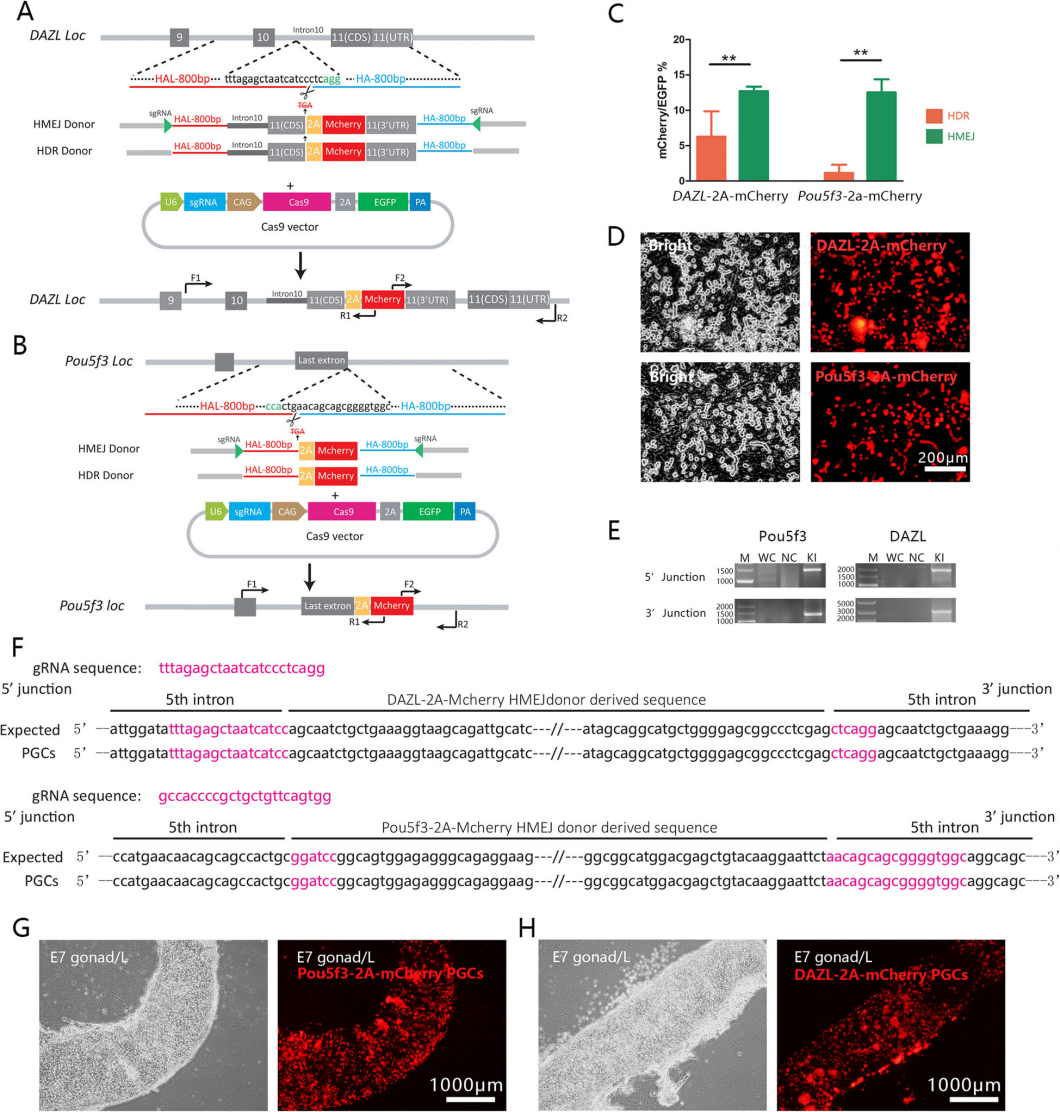
HMEJ mediates robust gene integration in chicken PGCs. **a** and **b** Plasmid design for gene integration at the *DAZL* and *Pou5f3* loci; **c** Efficiency of HITI, HDR and HMEJ gene integration at the *DAZL* and *Pou5f3* genes in PGCs; **d** PGCs bearing stable integration at *DAZL* and *Pou5f3*; **e** PCR of the site-specific gene integration in *DAZL*-2A-mCherry and *Pou5f3*-2A-mCherry cell lines; **f** Sequencing analysis of site-specific gene integration; G and H. The gonad migration of *Pou5f3*-2A-mCherry (**g**) and *DAZL*-2A-mCherry (**h**) PGCs were observed in 7 days embryos

To test our CRISPR/Cas9 gene integration system in chicken primordial germ cells (PGCs), the germ cell-specific gene *DAZL* and the pluripotency-related gene *Pou5f3* were targeted (Fig. 3a and b). Off-target analyses of the sgRNAs were performed and reveal their specific for *DAZL* and *Pou5f3* gene (Additional file 1: Figure S1B, C). Donor plasmids were designed using different strategies and were subsequently co-transfected with CRISPR/Cas9 plasmids into PGCs. Three days after transfection, EGFP positive cells were sorted and cultured for an additional 4 days. Analysis of the gene integration at the *DAZL* locus, based on the number of mCherry positive cells, represented a 6.25 and 12.7% knock-in efficiency for HDR and HMEJ constructs, respectively (Fig. 3c). At the *Pou5f3* locus, the gene integration efficiency was 0 and 12.5% for HDR and HMEJ, respectively (Fig 3c). Significant differences between the three different strategies were found at the *DAZL* and *Pou5f3* loci. The data suggested that even though the site-specific gene integration efficiencies of different strategies in different cell and gene targets were variable, HMEJ represented the most consistent and reliable strategy for gene targeted integration in PGCs. PGCs bearing *DAZL*-2A-mCherry and *Pou5f3*-2A-mCherry established by HMEJ (Fig. 3d) represented accurate gene integrations at targeted gene loci (Fig. 3e, f).

The abilities to migrate and colonize the embryonic gonad are some of the unique characteristics of PGCs. To evaluate the migration potential of PGCs labeled with *DAZL*-mCherry and *Pou5f3*-mCherry, these cells were injected into chicken embryos incubated to stage HH15. The migration of these cells to embryonic gonads was then examined 5 days after cell transplantation. Based on fluorescence microscopy, mCherry positive cells were observed in both of the gonads in the embryos (Fig. 3g and h).

### mCherry does not disturb gene expression and typical germ cell characteristics of PGCs

**Fig. 4:**
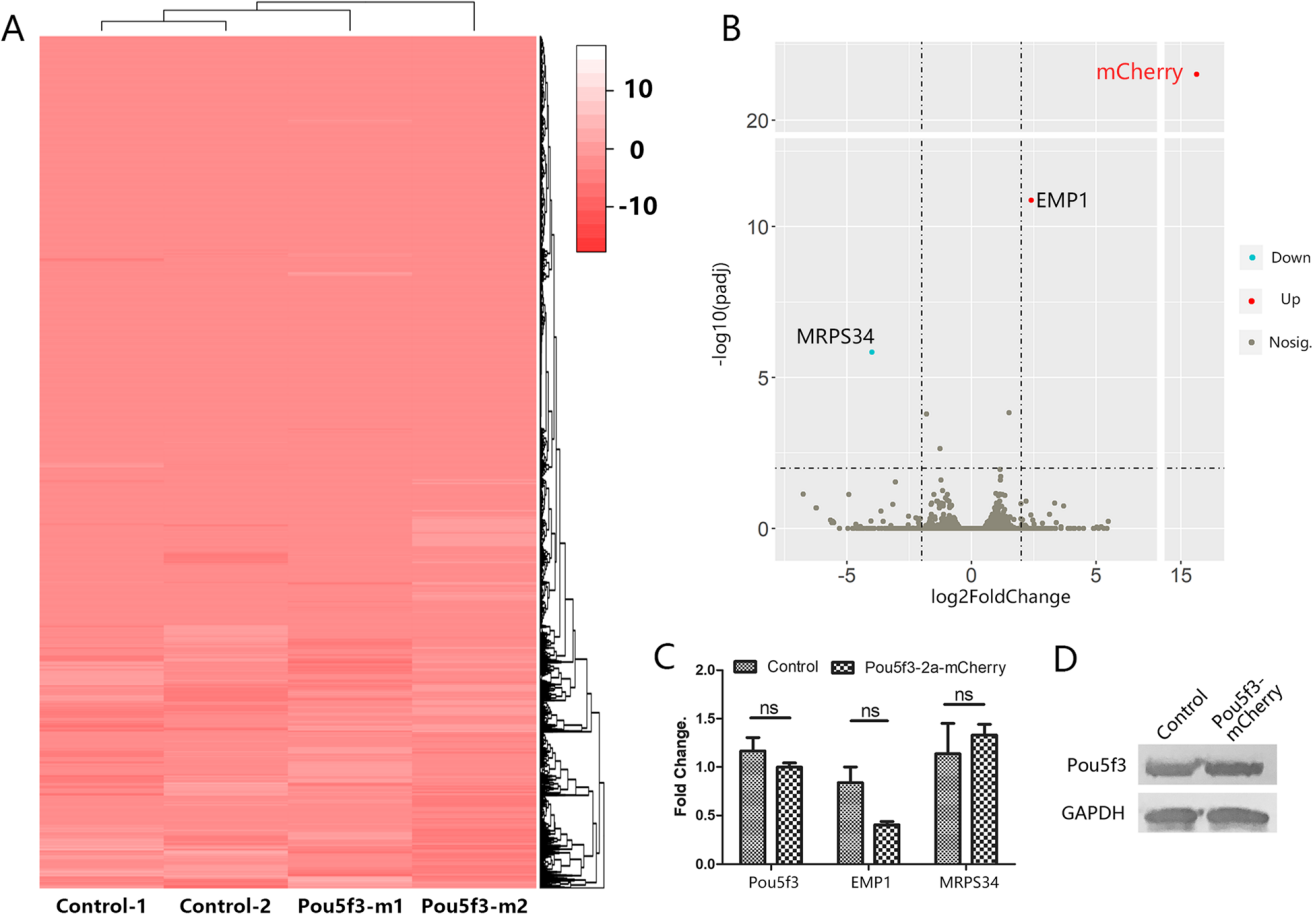
Gene expression analysis of *Pou5f3*-mCherry labeled and unlabeled PGCs. **a** Heatmap and hierarchical clustering of genes expressed in unlabeled (Control) and mCherry-labeled (*Pou5f3*-m) PGCs; **b** Volcano map of differentially expressed genes between Control and mCherry-labeled groups; **c** Q-PCR verification of the differentially expressed genes between Control and *Pou5f3*-mCherry cell line; **d** Western blots of *Pou5f3* protein for Control and *Pou5f3*-mCherry cell lines

In order to determine the effect of gene integration in *Pou5f3*-mCherry labeled PGCs, transcriptomic analysis was performed. A total of 1 × 10^6^ labeled and unlabeled PGCs were collected and snap frozen for RNA sequencing. Sequencing data was subjected to mapping (Hisat2), quantification (HTseq) and differential gene expression profiling (DEseq2). Except for the genes EMP1 and MRPS34, there were no obvious differences in gene expression patterns in the RNA sequencing results from *Pou5f3*-mCherry labeled or non-labeled PGCs (Fig. 4a and b). These two genes showed no significant expression differences by Q-PCR (Fig. 4c). After being mCherry labeled, PGCs showed similar *Pou5f3* expression at the RNA and protein levels (Fig. 4c and d). This result suggested that these PGCs maintained their germ-related and pluripotent characteristics after gene editing.

### Site-specific gene integration at the chicken OVAL gene

**Fig. 5:**
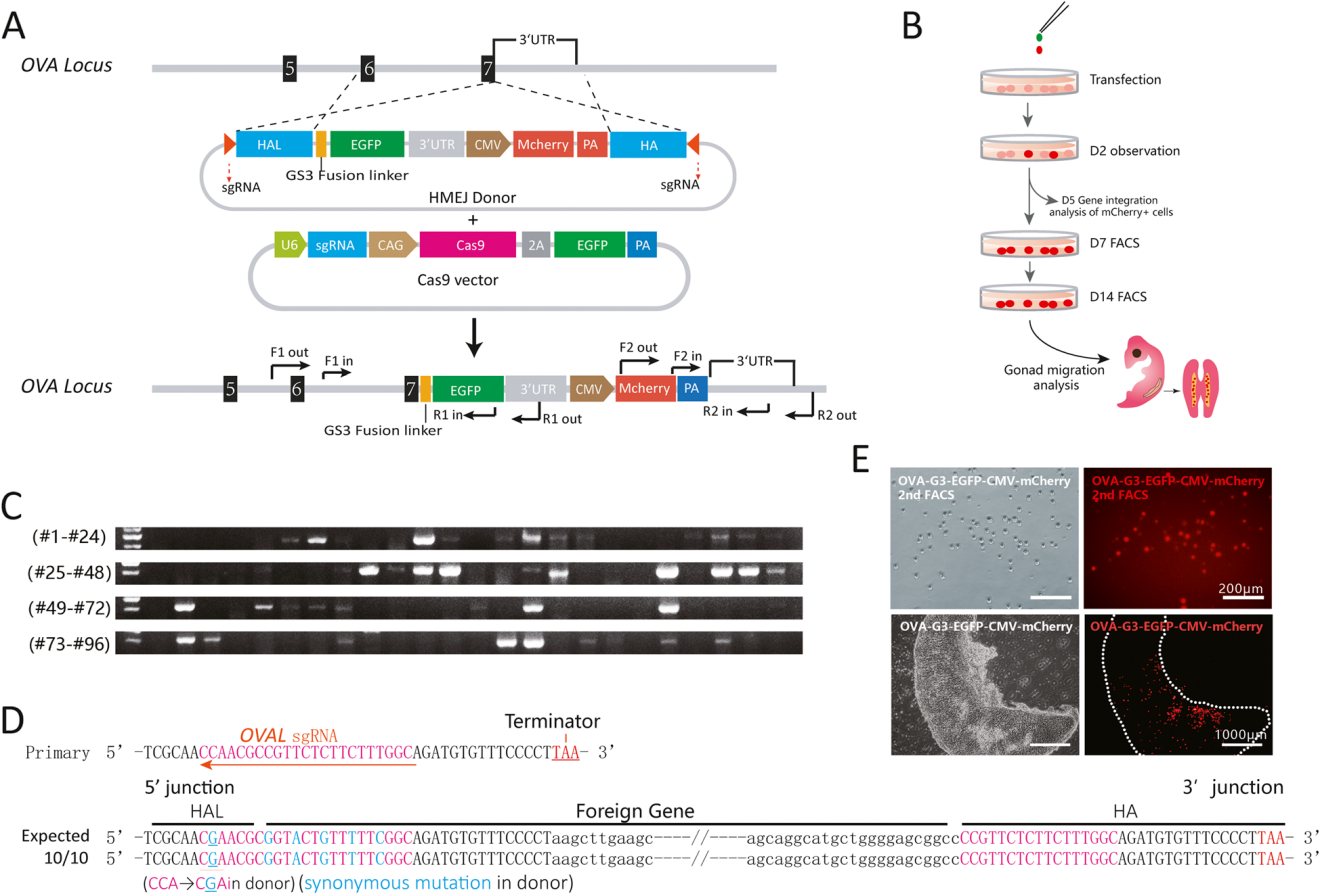
HMEJ-mediated ovalbumin gene modification in chicken PGCs. **a** Plasmid design for gene integration at the ovalbumin locus; **b** Experimental scheme for ovalbumin gene modification in PGCs; **c** Single cell PCR analysis of gene integration at the ovalbumin gene; **d** Sequencing analysis of site-specific gene integration at the ovalbumin gene; **e** Sequential FACS of OVAL modified PGCs (OVA-GS3-EGFP-CMV-mCherry) and their gonad migration in 7 days embryos

Since HMEJ proved to be efficient and reliable in chicken PGCs, we utilized this strategy to integrate a foreign gene at the chicken ovalbumin gene. An EGFP protein was fused to the end of the CDS of the ovalbumin gene, and an ectopic 5′ UTR region of the OVAL gene was linked to the end of the EGFP transcript. To make the gene integration in PGC visible, we inserted a mCherry protein regulated by a CMV promoter in the Donor plasmid (Fig. 5a). Five days after co-transfection of Cas9 and Donor plasmids, mCherry positive PGCs were picked for gene integration analysis (Fig. 5b). Single cell PCR of mCherry positive PGCs indicated a 30% OVAL gene integration efficiency (Fig. 5c). DNA sequencing revealed that these integrations happen in *DAZL/Pou5f3/OVAL* locus were accurate (Fig. 5d). After two rounds of FACS was performed at day 7 and day 14 after transfection, *OVAL*-fusion-*EGFP* PGC lines were established for further use. To evaluate the migration potential of *OVAL* modified PGCs, we injected these cells into chicken embryos recipients (stage HH15). Five days after injection, mCherry positive cells were observed in both of the gonads in the embryos (Fig. 5e). This result demonstrated that OVAL modified PGCs maintained their migration ability.

## Discussion

This study demonstrated that HMEJ mediated by CRISPR/Cas9 is a robust and efficient strategy for targeted gene integration in chicken cells. Precise gene knock-in mediated by HMEJ at the 3′ end of the *Pou5f3* gene showed no obvious influence on global gene expression or germ cell characteristics in chicken PGCs, suggesting its usefulness for developmental biology and gene function research. Moreover, HMEJ mediated gene integration holds good promise for gene editing in chicken, particularly to produce proteins utilizing endogenous gene regulation systems.

Foreign gene integration in chicken is typically achieved using lenti-virus or plasmid vectors with random and multi-copy insertions, which may cause genetic changes and unpredictable consequences in the derived animals [12, 13, 27–29]. The conditional expression of an exogenous gene is often required for the specific purpose and this is commonly achieved by using a tissue specific promoter [30, 31]. However, transgene expression driven by a foreign promoter cannot fully mimic endogenous gene expression, as some of the regulatory elements are absent in inserted expression cassettes or expression cassettes can be subjected to position effects [12, 32]. Thus, site-specific gene integration using nucleases are a potential strategy for avoiding the above-mentioned issues.

A robust and efficient gene integration strategy is necessary for chicken gene function elucidation and utilization. In chicken, gene integration mediated by CRISPR/Cas9 has been achieved via NHEJ and HDR [2, 33]. However, in non-dividing or slowly dividing cells, particularly in some primary cell types, gene integration is difficult to achieve by NHEJ or HDR [24]. Additionally, HITI-mediated gene integration is based on the NHEJ DNA repair mechanism and had poor gene integration efficiency in DF-1 cells in our study. HDR had good integration efficiency in DF-1 cells but variable efficiency in PGCs (Figs. 2 and 3). This could have been due to the lower dividing potential and suspension culture requirements of PGCs as compared to DF-1 cells. Gene integration meditated by HMEJ, a strategy utilizing both the homologous arm and double strand break-repairing mechanisms, showed consistent gene integration efficiency in both DF-1 and PGCs. This consistent efficiency of HMEJ indicated its applicability in the gene modification of chicken PGCs, which is the most important cell type for use in transgenic animal production in this species [5, 34].

Site-specific integration of foreign genes under endogenous promoters or as fusion with an endogenous protein has the advantage of utilizing endogenous gene regulatory systems. In the research of gene function, gene labeling using a fused fluorescent protein should be a reliable way to reveal gene expression patterns. In the present study, mCherry fused to the endogenous *DAZL/Pou5f3* gene yielded visualization of synchronized gene expression in PGCs. DAZL and Pou5f3 correspond to the characteristics of germ-related and pluripotency of PGCs, respectively. The successful endogenous labeled of these gene would be helpful to track the PGCs self-renew and differentiation processes in vitro or in vivo in the future. Importantly, the site-specific gene integration mediated by HMEJ did not disturb global gene expression profiles as seen in our transcriptomic analysis of *Pou5f3* labeled PGCs. Significant migration to embryonic gonads was also seen in these cells after transplantation to early embryos, suggesting the maintenance of germ cell characteristics after transplantation and yielding promise for use in the production of transgenic chicken and developmental biology research. Moreover, with regards to protein drug generation using a bioreactor, the ability to utilize the regulatory machinery of an endogenous gene with abundant expression could potentially lead to higher production yields of proteins of interest. In this study, we successfully inserted the EGFP gene at the end of the ovalbumin mediated by the HMEJ strategy. Since ovalbumin protein accounts for more than 60% of proteins in chicken eggs, this strategy holds good promise for the mass production of protein with pharmaceutical importance using the chicken oviduct system.

Due to its unique embryonic development in vitro and the efficiency of egg production due to the short generation interval of chickens, the chicken is an excellent model for use in developmental biology and for bioreactors for protein drug production [35]. Based on the robust and efficient gene integration demonstrated in this study, the HMEJ strategy can facilitate the elucidation of target gene function in biology and diseases as well as accelerate the use of the abundant genetic resources of chicken.

## Conclusion

This work demonstrated that HMEJ efficiently mediates site-specific gene integration in chicken PGCs. The successfully application of HMEJ strategy in *DAZL*, *Pou5f3* and *Ovalbumin suggested its* great potential for the target gene function elucidation and biopharmaceutical engineering.

## Methods

### Construction of plasmids

For CRISPR/Cas9 induced DNA cleavage in chicken cells, U6-gRNA components were introduced into the plasmid carrier pHEf1A9-mKate (Hesheng Bio-tek, Beijing, China), and co-transfected into DF-1 cells with an EYFP (enhance yellow fluorescence protein) truncated plasmid (6μg, 1:1). For Endogenous *DAZL* gene knock out, we constructed the plasmid pHEf1A9-hCas9-2A-EGFP for tracking the expression of the Cas9 protein, and a U6-sgRNA-pHEf1A9-mKate plasmid to track the gRNA. For gene integration, an all-in-one vector px458 (Addgene catalog no.48138), carrying the U6-gRNA-CAG-Cas9-2A-EGFP cassette, was used in later experiments. Plasmid px458 was linearized and ligated with oligos for introducing targeting sites.

To construct a HITI donor for *ACTB*, donor DNA (*actb*-2A-mCherry) was sandwiched between 23 nt *ACTB*-sgRNA target sequences (same direction) and then subcloned into a pMD18-T vector (Takara Biomedical Technology, Dalian, China).

To construct the HDR donor for *ACT*B, an 800 bp fragment of DNA around an *ACTB* targeted position was first cloned. Donor DNA (*actb*-2A-mCherry) was then ligated with 800 bp homology arms on both sides, and then subcloned into a pMD18-T plasmid (Takara Biomedical Technology, Dalian, China).

For the HMEJ donor *ACTB*, the cassette from the *ACTB*-HDR donor (HAL-*actb*-2A-mCherry-HA) was sandwiched by 23 nt *ACTB*-sgRNA target sequences (different directions) and then subcloned.

HITI, HDR, HMEJ donor plasmids for *DAZL*, *Pou5f3* and Ovalbumin were all constructed similarly to those of *ACTB*. Briefly, donor DNA (*DAZL*-2A-mCherry for *DAZL*, 2A-mCherry for *Pou5f3*, and 2A-EGFP-3’UTR-CMV-mCherry-PA) was sandwiched by 23 nt target sequences (same direction) and subcloned into a PMD18T vector as a HITI donor. Homology arms were added to both sides of the donor DNA from *DAZL* and *Pou5f3*. For HMEJ donors, the HDR cassette was sandwiched by 23 nt target sequences (different directions) and then subcloned.

The resulting fragments were purified with a Gel Extraction Kit (Tiangen Biotech, Beijing, China). All the plasmids were purified using a Plasmid Midi Kit (Omega Bio-Tek Inc., Norcross, GA, USA) and verified by Sanger sequencing.

### Cell culture and transfection

DF-1 cells were cultured in DMEM/F12 medium containing 10% fetal bovine serum (FBS). The PGCs used were derived from a domestic chicken species (three-yellow chicken) and stored in our lab [5, 36]. Chicken PGCs were cultured in a cKO medium composed of KO-DMEM, supplemented with 7.5% fetal bovine serum (FBS; Hyclone, USA), 20% BRL conditioned medium, 2.5% chicken serum (Sigma, St. Louis, MO, USA), 2 mM glutamine, 1 mM pyruvate, 1× nucleosides, 1× non-essential amino acids, 0.1 mM β-mercaptoethanol and 4 ng/mL human recombinant FGF. All cells were cultured at 37 °C in a 5% CO_2_ environment.

DF-1 cells and PGCs were transfected in the presence of Lipofectamine 3000 Reagent according to the manufacturer’s instructions. Briefly, a total 8 μg of plasmids (Cas9: donor = 1: 1) were used for each well of a six-well plate, and the transfection solution was removed 12 h after transfection. Positive cells were sorted 3 days after transfection by FACS for further culture and analysis. For stable PGC line establishment, a second FACS was performed 14 days after the first sorting. All components without special statements were bought from ThermoFisher (ThermoFisher Scientific, Waltham, MA, USA).

### T7 endonuclease I assay

DNA extraction was performed using a DNA extraction kit (Tiangen Biotech, Beijing, China). A T7E1 assay to detect genetic alterations was performed according to the manufacturer’s directions (NEB, Beijing, China). A nest-PCR priming the targeted position was performed the before the T7 endonuclease I (T7E1) assay. After digestion of the PCR product for 15 min, targeted gene mutations were observed by running a 2% agarose gel. Gray value analysis was done using Image J software.

### Gene integration analysis

For gene integration analysis, DF-1 and PGCs cells were collected, washed twice in PBS (phosphate buffer saline) and analyzed using a BD C6 flow cytometer. The subsequent data was analyzed using FlowJo software V10.

For DF-1 cells, DNA fragments around targeted sites were cloned into the pMD18-T (Takara Biomedical Technology, Dalian, China) vector for gene knock out analysis. In the transferred bacteria, 10 clones were randomly picked for DNA sequencing. For site-specific gene integration verification of the *ACTB* locus, single cells were picked based on mCherry fluorescence and transferred into a PCR tube containing lysis buffer (0.1% tween 20, 0.1% Triton X-100 and 4 μg/mL proteinase K). After incubation for 30 min at 56 °C and heat inactivation of the proteinase K at 95 °C for 5 min, the samples were then used for nest-nest PCR analysis. The products of the second PCR were gel purified and Sanger sequenced.

For established *DAZL*-2A-mCherry and *Pou5f3*-2A-mCherry PGCs cell lines, total DNA was directly extracted from cells for PCR and Sanger sequencing.

### Off-targeted analysis

The off-target sites were predicted online by ChopChop (https://chopchop.cbu.uib.no/). The top 4 off-target sites were chosen to test the off-target effect in different gene modified cell lines. After DNA extraction, PCR amplification for different target sites in different cell lines was performed. The PCR products of the second PCR were gel purified and Sanger sequenced.

### Q-PCR analysis

Total RNA was extracted from PGCs using the Omega RNA kit. The first-strand cDNA was synthesized using a Reverse Transcription Reagent Kit and gDNA Eraser (Takara Biomedical Technology, Dalian, China) was used to remove contaminating genomic DNA. Q-PCR reactions were performed using TB Green Premix Ex Taq II (Takara Biomedical Technology, Dalian, China).

### Western blot analysis

Total proteins from different samples were extracted using RIPA lysis buffer (China, Solarbio, cat. R0020). Antibodies against GAPDH (Abcam, Cambridge, MA, USA; 1:3000, 4 °C overnight) and Pou5f3 (Santa Cruz Biotechnology, Dallas, USA; 1:500, 4 °C overnight), as well as a secondary antibodies labeled with AP (ZSGB-Bio, Beijing, China; room temperature, 1 h), were used for Western blots. A BCIP/NBTAP chromogenic Kit (Beyotime Biotec, Shanghai, China) was used to visualize proteins. Gray value analysis was completed using Image J software.

### RNA sequencing

Approximately 1 × 10^6^ PGCs (or more) were harvested for total RNA, washed twice using PBS and then snap frozen in liquid nitrogen. Total RNA was extracted using the RNeasy Plus Mini kit from QIAGEN (QIAGEN, Shanghai, China). Standard mRNA libraries were prepared using the NEBNext II Ultra Directional RNA Library Prep kit from England Biolabs (NEB, Beijing, China) and sequenced on an Illumina NextSeq500. RNA sequencing data are available in NCBI (SRR10058581-SRR10058584).

Sequenced reads were aligned to the chicken genome using Hisat2. The mapping results were quantified across all gene exons using HTseq [30, 37], and differential gene expression was carried out with DESeq2 v1.14.1 [30] using two replicates to compute within-group dispersion and to compare and contrast between gene-integrated and non-integrated conditions.

### PGC migration assays

A total of 10,000 PGCs labeled with mCherry were injected into stage HH15 chicken embryos. After 5 days of incubation, embryonic gonads were isolated and observed with a fluorescence microscope.

## Supplementary information

**Additional file 1: Figure S1.** The off-target analysis of *ACTB*(A), *DAZL*(B), *Pou5f3*(C) and *OVAL*(D) potential off-target locus. No overlapping peaks reveal there was no off-target effect in predicted off-target locus in cell lines genome.

## Data Availability

Not applicable.

## Abbreviations

DSB: Double strand break
EGFP: Enhance green fluorescence protein
EYFP: Enhance yellow fluorescence protein
FBS: Fetal bovine serum
HDR: Homology-directed repair
HITI: Homology-independent targeted integration
HMEJ: Homology mediated end joining
PBS: Phosphate buffer saline
PGCs: Primordial germ cells
T7EI: T7 endonuclease I

## References

[1] Goldman I, Kadulin S, Razin S (2004). Transgenic animals in medicine: integration and expression of foreign genes, theoretical and applied aspects. Med Sci Monit.

[2] Taylor L, Carlson DF, Nandi S, Sherman A, Fahrenkrug SC, McGrew MJ (2017). Efficient TALEN-mediated gene targeting of chicken primordial germ cells. Development.

[3] Park TS, Han JY (2012). piggyBac transposition into primordial germ cells is an efficient tool for transgenesis in chickens. Proc Natl Acad Sci U S A.

[4] Yao X, Wang X, Hu X, Liu Z, Liu J, Zhou H (2017). Homology-mediated end joining-based targeted integration using CRISPR/Cas9. Cell Res.

[5] Xie L, Lu Z, Chen D, Yang M, Liao Y, Mao W (2019). Derivation of chicken primordial germ cells using an indirect co-culture system. Theriogenology..

[6] Hofmann A, Kessler B, Ewerling S, Kabermann A, Brem G, Wolf E (2006). Epigenetic regulation of lentiviral transgene vectors in a large animal model. Mol Ther.

[7] Laible G, Wei J, Wagner S (2015). Improving livestock for agriculture–technological progress from random transgenesis to precision genome editing heralds a new era. Biotechnol J.

[8] Van Reenen C, Meuwissen T, Hopster H, Oldenbroek K, Kruip TA, Blokhuis HJ (2001). Transgenesis may affect farm animal welfare: a case for systematic risk assessment. J Anim Sci.

[9] Semplerowland SL, Berry J (2014). Use of lentiviral vectors to deliver and express bicistronic transgenes in developing chicken embryos. Methods.

[10] Motono M, Yamada Y, Hattori Y, Nakagawa R, Nishijima K, Iijima S (2010). Production of transgenic chickens from purified primordial germ cells infected with a lentiviral vector. J Biosci Bioeng.

[11] Tan W, Proudfoot C, Lillico SG, Whitelaw CBA (2016). Gene targeting, genome editing: from Dolly to editors. Transgenic Res.

[12] Dimitrov L, Pedersen D, Ching KH, Yi H, Collarini EJ, Izquierdo S (2016). Germline gene editing in chickens by efficient CRISPR-mediated homologous recombination in primordial germ cells. PLoS One.

[13] Kobayashi T, Kato-Itoh M, Yamaguchi T, Tamura C, Sanbo M, Hirabayashi M (2012). Identification of rat Rosa26 locus enables generation of knock-in rat lines ubiquitously expressing tdTomato. Stem Cells Dev.

[14] Kim Y-G, Cha J, Chandrasegaran S (1996). Hybrid restriction enzymes: zinc finger fusions to Fok I cleavage domain. Proc Natl Acad Sci U S A.

[15] Porteus MH, Baltimore D (2003). Chimeric nucleases stimulate gene targeting in human cells. Science.

[16] Hockemeyer D, Wang H, Kiani S, Lai CS, Gao Q, Cassady JP (2011). Genetic engineering of human pluripotent cells using TALE nucleases. Nat Biotechnol.

[17] Ran FA, Hsu PD, Wright J, Agarwala V, Scott DA, Zhang F (2013). Genome engineering using the CRISPR-Cas9 system. Nat Protoc.

[18] Cong L, Ran FA, Cox D, Lin S, Barretto R, Habib N (2013). Multiplex genome engineering using CRISPR/Cas systems. Science.

[19] Hsu PD, Lander ES, Zhang F (2014). Development and applications of CRISPR-Cas9 for genome engineering. Cell.

[20] Wang B, Li K, Wang A, Reiser M, Saunders T, Lockey RF (2015). Highly efficient CRISPR/HDR-mediated knock-in for mouse embryonic stem cells and zygotes. Biotechniques.

[21] Lee JS, Kallehauge TB, Pedersen LE, Kildegaard HF (2015). Site-specific integration in CHO cells mediated by CRISPR/Cas9 and homology-directed DNA repair pathway. Sci Rep.

[22] Orthwein A, Noordermeer SM, Wilson MD, Landry S, Enchev RI, Sherker A (2015). A mechanism for the suppression of homologous recombination in G1 cells. Nature.

[23] Cox DBT, Platt RJ, Zhang F (2015). Therapeutic genome editing: prospects and challenges. Nat Med.

[24] Suzuki K, Tsunekawa Y, Hernandez-Benitez R, Wu J, Zhu J, Kim EJ (2016). In vivo genome editing via CRISPR/Cas9 mediated homology-independent targeted integration. Nature.

[25] Van Eenennaam A, Young A (2014). Prevalence and impacts of genetically engineered feedstuffs on livestock populations. J Anim Sci.

[26] Mahdi E, Fariba K (2012). Overview on the efforts to generate transgenic chicken. Afr J Biotechnol.

[27] Oishi I, Yoshii K, Miyahara D, Kagami H, Tagami T (2016). Targeted mutagenesis in chicken using CRISPR/Cas9 system. Sci Rep.

[28] Coleman CM (2008). Chicken embryo as a model for regenerative medicine. Birth Defects Res C Embryo Today.

[29] Norris AL, Lee SS, Greenlees KJ, Tadesse DA, Miller MF, Lombardi H. Template plasmid integration in germline genome-edited cattle. BioRxiv. 2019;715482. 10.1038/s41587-019-0394-6 32034391

[30] Lillico S, Sherman A, McGrew M, Robertson C, Smith J, Haslam C (2007). Oviduct-specific expression of two therapeutic proteins in transgenic hens. Proc Natl Acad Sci U S A.

[31] Houdebine LM (2009). Production of pharmaceutical proteins from transgenic animals. J Biotechnol.

[32] Gasiunas G, Barrangou R, Horvath P, Siksnys V (2012). Cas9–crRNA ribonucleoprotein complex mediates specific DNA cleavage for adaptive immunity in bacteria. Proc Natl Acad Sci U S A.

[33] Lee Hong Jo, Yoon Jong Won, Jung Kyung Min, Kim Young Min, Park Jin Se, Lee Kyung Youn, Park Kyung Je, Hwang Young Sun, Park Young Hyun, Rengaraj Deivendran, Han Jae Yong (2019). Targeted gene insertion into Z chromosome of chicken primordial germ cells for avian sexing model development. The FASEB Journal.

[34] Van de Lavoir M-C, Diamond JH, Leighton PA, Mather-Love C, Heyer BS, Bradshaw R (2006). Germline transmission of genetically modified primordial germ cells. Nature.

[35] Lillico SG, Mcgrew MJ, Sherman A, Sang HM (2005). Transgenic chickens as bioreactors for protein-based drugs. Drug Discov Today.

[36] Wang L, Chen M, Chen D, Peng S, Zhou X, Liao Y (2017). Derivation and characterization of primordial germ cells from Guangxi yellow-feather chickens. Poult Sci.

[37] Pertea M, Kim D, Pertea GM, Leek JT, Salzberg SL (2016). Transcript-level expression analysis of RNA-seq experiments with HISAT, StringTie and Ballgown. Nat Protoc.

