# Supplementary figures and images for: HMEJ-mediated efficient site-specific gene integration in chicken cells
Source: J Biol Eng. 2019 Nov 21;13:90. doi: 10.1186/s13036-019-0217-9 (PMC6868705; doi:10.1186/s13036-019-0217-9)

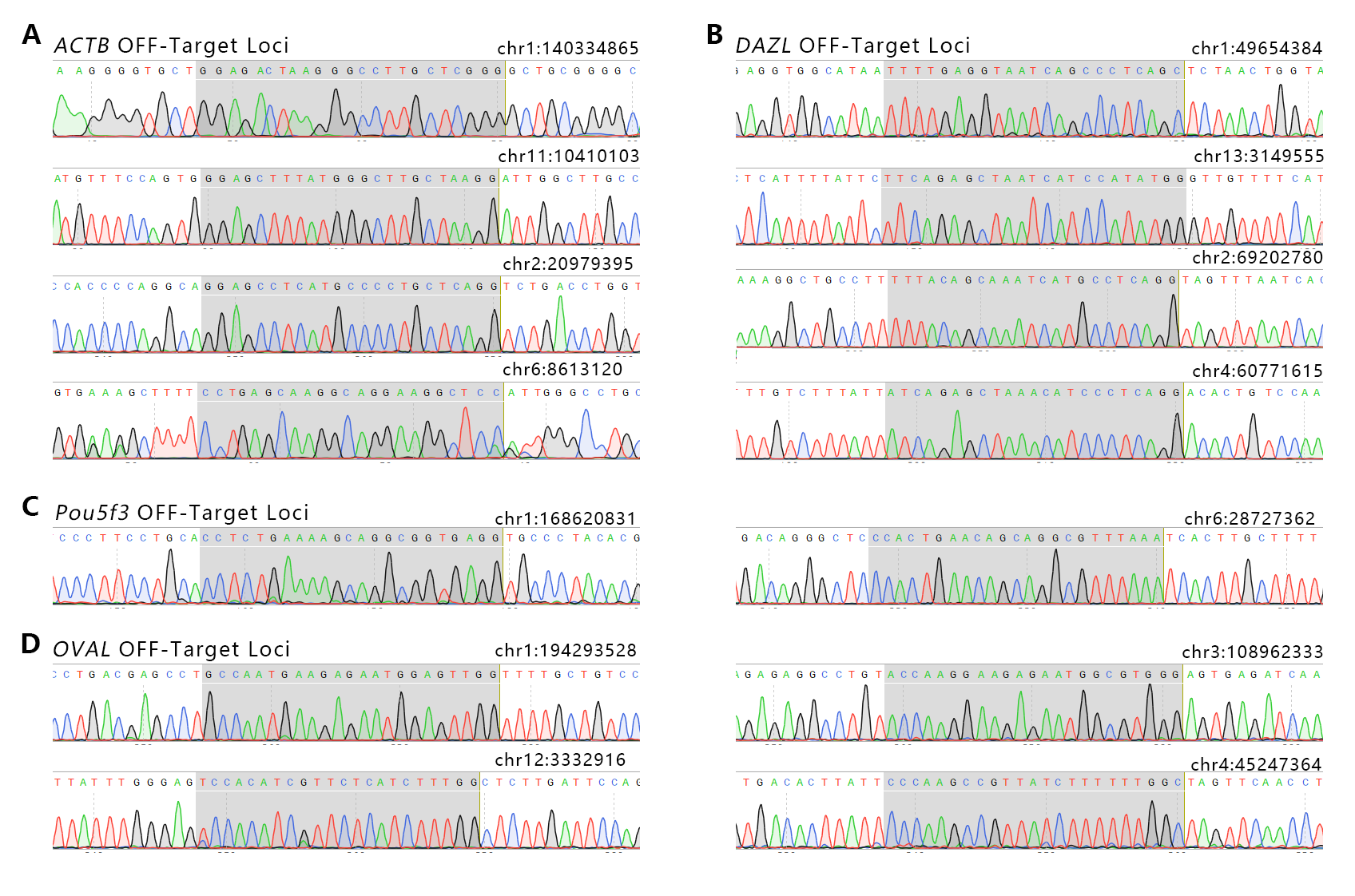

Supplement: Supplementary file 1 — Additional file 1: Figure S1. The off-target analysis of ACTB(A), DAZL(B), Pou5f3(C) and OVAL(D) potential off-target locus. No overlapping peaks reveal there was no off-target effect in predicted off-target locus in cell lines genome. [file 13036_2019_217_MOESM1_ESM.tif]
